# Relationship between Endplate Defects, Modic Change, Disc Degeneration, and Facet Joint Degeneration in Patients with Low Back Pain

**DOI:** 10.1155/2019/9369853

**Published:** 2019-07-11

**Authors:** Bin Lv, Jishan Yuan, Hua Ding, Bowen Wan, Qinyi Jiang, Yongjun Luo, Tao Xu, Peng Ji, Yilei Zhao, Lei Wang, Yan Wang, Anquan Huang, Xiang Yao

**Affiliations:** ^1^Department of Orthopedics, The Affiliated People's Hospital of Jiangsu University, Zhenjiang, Jiangsu Province 212002, China; ^2^Department of Orthopedics, The First Affiliated Hospital of Nanjing Medical University, Nanjing, Jiangsu Province 210029, China; ^3^Department of Orthopedics, The Affiliated Suzhou Hospital of Nanjing Medical University, Suzhou Municipal Hospital, Suzhou, Jiangsu Province 215002, China

## Abstract

**Purpose:**

The endplate defects (EDs), Modic changes (MCs), disc degeneration (DD), facet orientation (FO), and facet tropism (FT) were demonstrated to be related to the low back pain (LBP). The aim of this study was to investigate possible correlations between them.

**Methods:**

75 patients were reviewed to evaluate the degenerative change in vertebral bodies (EDs and MCs), intervertebral discs (DD), and facet joint degeneration (FO and FT). All patients were categorized into four groups based on the grade of EDs. Clinical outcomes were evaluated with the visual analog scale (VAS) and Oswestry disability index (ODI) before and after surgery.

**Results:**

There was no difference between the four groups in baseline characteristics except for gender and weight. FT is positively correlated with FO. The same rule exists between EDs, the size of MCs II, FO (left) and FO (right), and VAS and ODI. The grade of EDs is positively correlated with the grade of DD. L4-L5 can bear more load than other levels; thus, the grade of EDs is higher than that of other lumbar levels. The preoperative LBP was relieved in all groups in varying degrees. The change of pain and dysfunction is inversely proportional to the grade of EDs in the general trend.

**Conclusion:**

The relationship between weight, gender, and disc degeneration provided a mechanism by which increasing weight can predispose to DD. Different grades of EDs had different effects on patients with LBP. There was a significant correlation between EDs, MCs II, DD, FT, and FO.

## 1. Introduction

Low back pain (LBP), usually complained as pain accompanied by muscle stiffness with or without radiation to the lower extremity, is the disabling symptom with enormous impact on population health [1]. Conservative pain-relieving treatments such as medial branch blocks and intraarticular injections could offer short-term pain relief but did not treat degenerative tissues or underlying mechanisms. Percutaneous transforaminal endoscopic discectomy did not remove the facet joints to reduce pain, which prevents the risk of adjacent segment disease [2].

The intervertebral disc, the left facet joint, and the right facet joint form the ‘three-joint complex' connected in series. These three anatomical factors can affect each other [3]. Previous studies demonstrated that facet tropism was a potential pathogenic factor for the progression of disc degeneration and facet joint degeneration, which can cause or aggravate low back pain [4, 5]. Operation inevitably accelerated disc degeneration and facet joint degeneration in the operating segments. Consequently, patients may get different clinical outcomes. Percutaneous transforaminal endoscopic discectomy (PTED) had the advantages of small tissue damage in the treatment of LBP. However, it is unknown whether PTED could protect the vertebral body from endplate defects by preserving the facet joint.

MCs were fairly commonly observed in the lower lumbar spine. Edema and inflammation originating in type I MCs (low T1 and high T2 signals in MRI) play an important role in nonspecific chronic low back pain (Table 1). Besides, type II MCs (high T1 and T2 signals) indicate a progression to fatty involution and type III MCs (low T1 and T2 signals) indicate vertebral endplate bone sclerosis, respectively [6]. However, despite various theories, it is still unclear what is the exact pathogenesis underlying MCs [7]. MCs, DD, and facet joint degeneration (FJ) have proved to be present in patients with LBP, even in asymptomatic individuals [8–10]. MCs also showed a high association with LBP apart from spinal structures.

Endplate defects were categorized into four types according to the severity of the damage. The facet tropism and facet orientation were related to the presence of MCs, since MCs and facet tropism were common in the lower lumbar spine. Facet tropism was suggested to be an anatomical factor associated with the DD and facet joint degeneration in the spine. Further, there was a strong correlation between the severity of facet osteoarthritis and the severe DD. DD and FJ have also been considered to be the sources of LBP. Relationship between MCs, DD, and progressive grades of endplate defect was shown, but FJ degeneration was not taken into consideration. It is generally considered that early stages of DD could cause instability [11]. The prevalence of low back pain increased as the development of lumbar disc degeneration [12]. Endplate defects, which are seen adjacent to a healthy disc, are associated with degenerative changes such as Modic changes (MCs), disc degeneration (DD), and Schmorl's nodes. Recent research demonstrated that endplate defects could also induce LBP. In contrast, endplate lesions caused by microfracture received less attention.

Numerous studies have investigated the relationship between MCs, DD, and facet joint degeneration. However, the role of endplate defects in MCs, DD, and facet joint degeneration is not confirmed. Several theories have been suggested regarding the etiology of these conditions, but the pathophysiology of endplate defect is still unclear. Therefore, we hypothesized that there would be an association between endplate defects, MCs, and facet joint degeneration in patients with LBP.

## 2. Patients and Methods

### 2.1. Patient Population

This retrospective study was approved by the institutional review board of Affiliated People's Hospital of Jiangsu University. A total of 322 patients who experienced one-level percutaneous transforaminal endoscopic discectomy between January 2014 and February 2018 were reviewed. The inclusion criteria were (1) lumbar disk herniation (LDH), suffering from LBP; (2) CT showing facet joint tropism and facet orientation; (3) MRI showing MCs type I, II; and (4) regular follow-up at 1, 2, 4, 8, 12, and 24 weeks after operation and 1 week before operation. A total of 75 patients met the inclusion criteria and were categorized into four groups (17 in the normal group, 29 in focal defects, 12 in corner defects group, and 17 in erosive defects group) based on endplate defects on MRI. Demographic data and surgical data were documented.

### 2.2. Assessment of DD

DD was quantitatively measured on mid-sagittal T2W images according to the previous description [13]. Disc signal intensity was acquired by defining a region of interest, as the disc measurement was further adjusted using the signal intensity of adjacent cerebrospinal fluid.

### 2.3. Assessment of Endplate Defects

**Figure 1:**
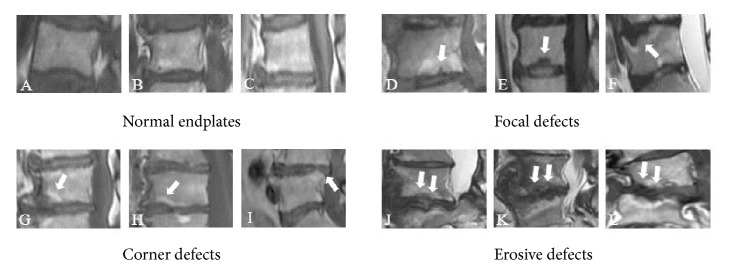
Morphological characteristics of three types of endplate defects. (A-C) Grade 0: normal endplates, no concave or defects. (D-F) Grade 1: endplate discontinuity or focal defects of the endplate (arrow). (G-I) Grade 2: defects located at the anterior or posterior corner of a vertebral body (arrow). (J-L) Grade 3: irregular and extensive disruptions of the endplate (arrow).

Endplate defects were visible as the loss or disruption of the complete appearance. Change of endplate defects visible on sagittal MR images was categorized into four grades according to Feng et al. [14]. Focal endplate defect was defined as a local hollow or discontinuity on the endplate, accompanied by nucleus protrusion into the subchondral bone. Corner endplate defect was defined as an anterior or posterior corner on the endplate, accompanied by local disrupted or absent vertebral trabeculae. An erosive endplate defect involved the extensive alteration of the endplate, such as an irregular, serrated, or worm-eaten appearance (Figure 1).

### 2.4. Assessment of MCs

MCs were categorized into type 0, I, II, and III using sagittal multipositional MRI image. Type 0 MCs showed normal endplate, type I MCs showed low T1 and high T2 signal, type II MCs showed high T1 and T2 signals, and type III MCs showed low T1 and T2 signals [15] (Table 1).

### 2.5. Assessment of Facet Joint Orientation and Facet Tropism

**Figure 2:**
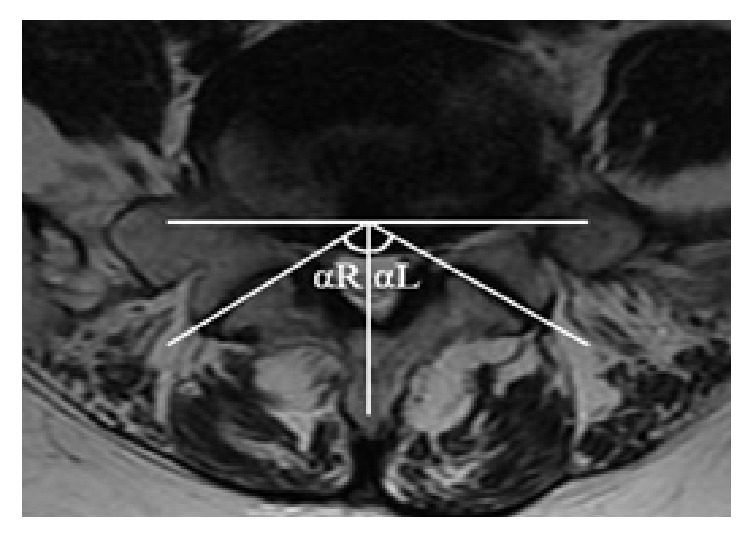
Evaluation of facet tropism and facet orientation. The facet joint angles relative to the sagittal plane were *α*L and *α*R. (a) Facet joint tropism = |*α*L − *α*R|. (b) Facet joint orientation = (*α*L +*α*R)/2.

The facet line was defined as the line connecting the two peaks of each of the superior articular facets. The angle between the facet line and the midsagittal line of the vertebra was defined as a facet angle (*α* is right facet angle, *β* is left facet angle). The facet joint orientation was evaluated as the mean value of the degree between the left facet angle and the right facet angle. The facet tropism was measured as the difference in degree between the left facet angle and the right facet angle (Figure 2).

### 2.6. Assessment of Facet Joints Osteoarthritis

**Figure 3:**
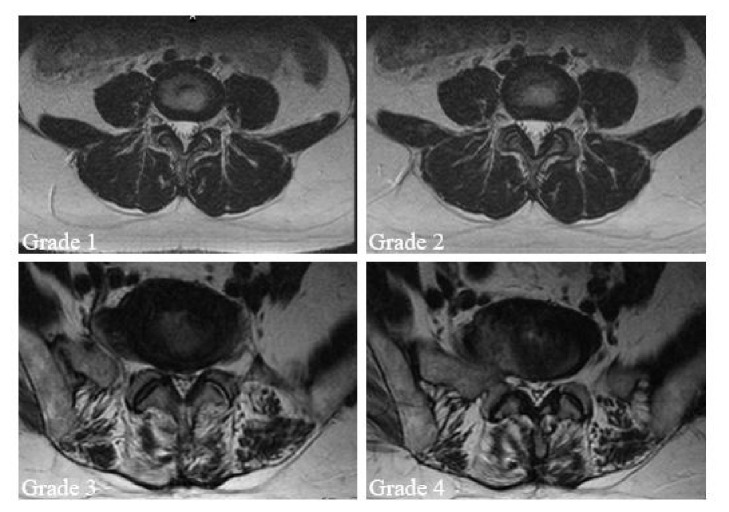
Facet osteoarthritis changes or degeneration was categorized into four grades using T2-weighted axial MRI. (a) Grade 1 was normal. (b) Grade 2 showed a narrow joint space and small osteophytes (mild osteoarthritis). (c) Grade 3 showed sclerosis or subchondral erosions (moderate osteoarthritis). (d) Grade 4 showed marked osteophyte (severe osteoarthritis).

Four grades of facet joint osteoarthritis were defined according to T2-weighted MRI [16]. Grade 1 was normal, Grade 2 facet joint osteoarthritis was presented as joint narrow space or mild osteophyte, Grade 3 facet joint osteoarthritis showed sclerosis or moderate osteophyte, and Grade 4 facet joint osteoarthritis was marked osteophyte (Figure 3).

### 2.7. Data Analysis

The outcome of imaging measurement was derived from the hospital's imaging system. All data were expressed as mean (SD) or mean (95%CI). Data analysis was done using SPSS 22.0 (IBM Corporation, USA). Multivariable analysis and Chi-square test for quantitative and qualitative variables, independent sample t-tests, and Spearman correlation test were used in our research. The result difference was statistically significant when* P*<0.05.

## 3. Results

### 3.1. Baseline Data and Clinical Outcomes

A total of 75 patients (38 men and 37 women) with a mean age of 55.8 ± 12.8 years were included and divided into four groups in the study. Baseline data of the four groups showed that age, height, occupation, alcohol consumption, smoking, BMI, duration of LBP, hypertension, diabetes mellitus, clinical outcomes, and lumbar level were similar among the four groups (P > 0.05). Gender and weight are significantly different among the four groups (P < 0.05) (Table 2).

### 3.2. Distributions for FJ Orientation and Facet Tropism in Four Types of Endplate Defect

**Figure 4:**
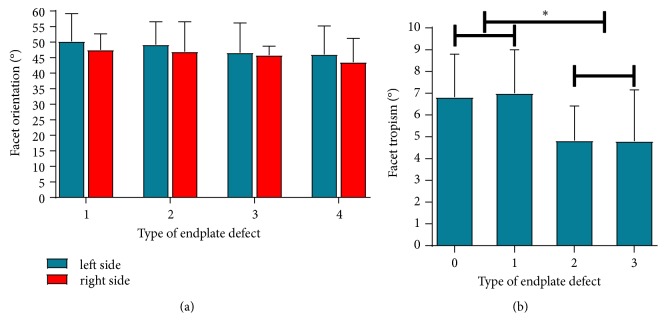
Distributions for facet orientation and facet tropism in four types of endplate defect. (a) Distributions for facet orientation in four types of endplate defect. (b) Distributions for facet tropism in four types of endplate defect. Note: *∗*P<0.05 between groups, the facet orientation was expressed as mean (SD), and facet tropism was expressed as mean (95%CI).

The severity of endplate defect was negatively related to the facet orientation in left side group and right side group, but the difference between the two groups was not statistically significant (P > 0.05). The facet tropism in type 0 endplate defect and type 1 endplate defect was greater than that in type 2 endplate defect and type 3 endplate defect (P < 0.05) (Figure 4).

### 3.3. Distribution of DD and Endplate Defect in the Four Groups

**Figure 5:**
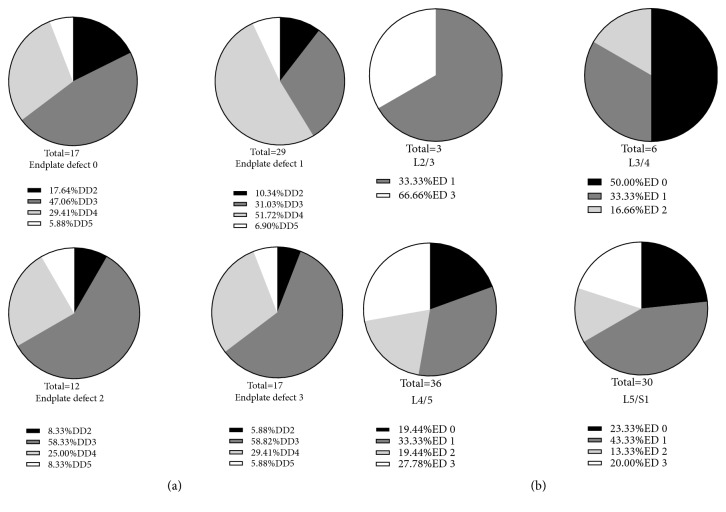
Endplate defects in association with Pfirrmann grades and intervertebral disc levels. (a) Comparison of distributions for DD on different grades of endplate defects. (b) Comparison of distributions for endplate defects on different levels. Abbreviations: ED: endplate defect; DD: disc degeneration.

150 adjacent endplates and 75 intervertebral discs from 75 patients were examined in our study. It is shown that the prevalence of Grade 3 disc degeneration (DD) was significantly higher than grade 2 DD, Grade 4 DD, and Grade 5 DD in endplate defect group 0, endplate defect group 2, and endplate defect group 3 (P<0.05, respectively) (Figure 2(a)). It is also shown that the prevalence of Grade 4 DD was significantly higher than Grade 2 DD, Grade 3 DD, and Grade 5 DD in endplate defect group 1(Figure 5(a)).

We found that DD mainly occurred at L2/3, L3/4 L4/5, and L5/S1 in patients treated with PTED. The prevalence of endplate defect in group 1 was higher than that in group 3 at L2/3. The prevalence of endplate defect in group 0 was higher than that in group 1 and 2 at L3/4. The prevalence of endplate defect in group 1 was the highest at L4/5 (33.33%), followed by group 3 (27.78%) and group 0 (19.45%), and the prevalence in group 2 was the lowest (19.44%). The prevalence of endplate defect in group 1 was the highest at L5/S1 (43.33%), followed by group 0 and 3 (23.33% and 20.00%), and the prevalence in group 2 was the lowest (13.33%)(Figure 5(b)).

### 3.4. Relationship between the Size of MCs II and the Degree of DD

**Figure 6:**
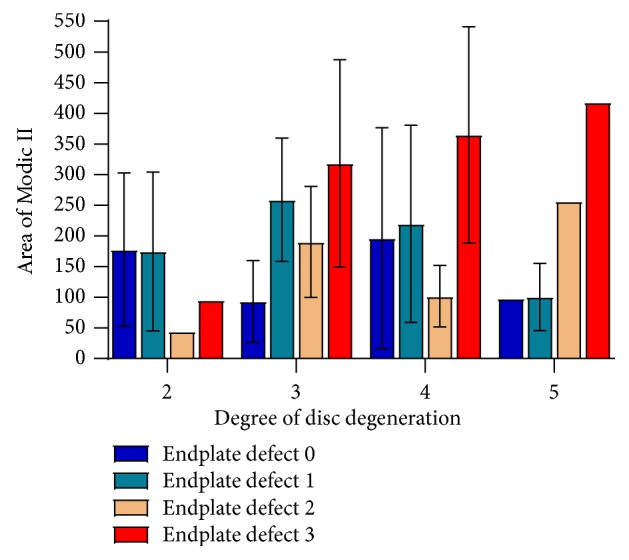
The relationship between the size of Modic II and the degree of DD at different types of endplate defects.

The endplate defect group 3 showed the largest size of MCs II in all degrees of DD except DD Grade 2. The sizes of MCs II in group 3 increased rapidly in DD Grade 2 and Grade 3 (Figure 6).

### 3.5. Correlation Outcomes

Pearson correlation analysis showed that there were positive correlations between FT (left) and FT (right) (r = 0.627, P<0.05), FJOA (left) and FJOA (right) (r = 0.581, P<0.05), FJOA (right) and DD(r = 0.230, P<0.05), and MCs II and FJOA (right) (r = 0.230, P<0.05) in patients with LBP, as well as the postoperative ODI score and VAS score(r = 0.740, P<0.05) (Table 3).

### 3.6. Clinical Outcomes

**Figure 7:**
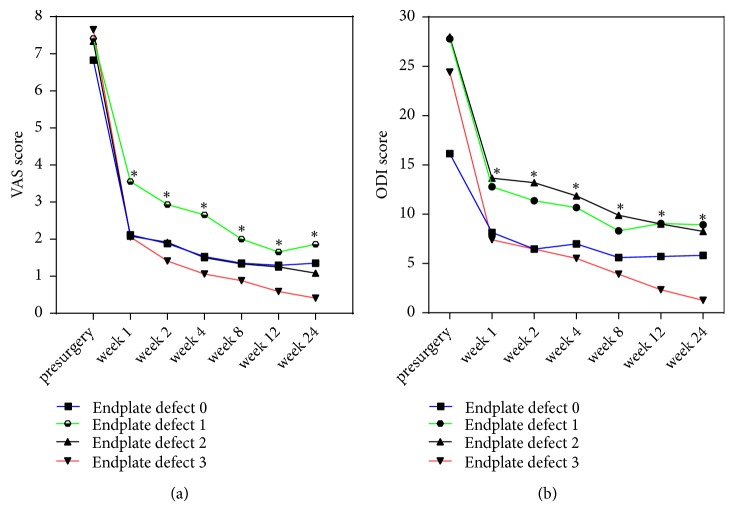
VAS scores (a) and ODI scores (b) before and after the operation. Abbreviations: VAS: visual analog scale; ODI: Oswestry disability index. Note: *∗*P<0.05 postsurgery vs presurgery in all groups.

The VAS score and ODI score were significantly different at the postoperative and preoperative time points (1, 2, 4, 8, 12, and 24 weeks after operation and 1 week before operation) in the four groups (P<0.05, respectively) (Table 4). The VAS score of group 3 was the lowest in all groups, followed by group 0 and group 2. The VAS score of group 1 was the highest. The largest decrease in ODI score was in group 3, followed by group 0. Group 1 and group 2 showed the slowest recovery rate at nearly all the time points. However, there was no statistically significant difference between the four groups by the end of the last follow-up (P>0.05) (Figure 7).

## 4. Discussion

Regarding LBP in patients with disc herniation, much attention is focused on DD, MCs, and FJ degeneration. Load distribution and FJ alignment were thought to be factors in the development and progression of facet osteoarthritis [17]. The FJs withstand the vast majority of shear forces and absorb 16% of the body load on average [18], while the disc bears the compression load. The ‘three-joint complex' in each segment is functionally interdependent, so changes in a separate structure affect the other structure. Another research has proven that FJs degeneration accelerated DD, which eventually lead to the occurrence of MCs [19]. Biomechanical research demonstrated that FJs allow the disc-joint to withstand loads, which indicates that disc torsion mechanics were dependent on the presence of FJs. Vernon et al. [20] demonstrated that DD leads to change of facet tropism and facet orientation, which is a risk factor for episodes of LBP [21]. However, we suggested that endplate defect, caused by microfracture in the vertebral body, would be an initiating factor for the clinical symptom. Our result was in accordance with recent reports [14]. To our knowledge, we are the first to evaluate the relationship between MCs, DD, and FJ degeneration in different types of endplate defects in patients with LBP. We investigated the distribution of endplate defects on different disc levels, DD grades, FJ orientation, and facet tropism. Patients with LDH have greater FJs orientation and more significant FJ tropism, which may lead to FJ osteoarthritis [16]. Consist with previous research, we found that FJ osteoarthritis is strongly associated with DD. As FJ osteoarthritis grade increases, worst postoperative clinical outcomes might be expected. However, our results did not find a significant correlation between FJ orientation and facet tropism in patients with LDH. This may be due to the mild disc herniation in patients undergoing PTED.

Previous studies demonstrated that endplate defects allow inflammatory cytokines, which pass from disc to the vertebral body, to initiate edema characteristically observed in MCs [22]. That endplate defects contribute to both MCs and DD owing to the physical location of the endplate, which was indirectly associated with FJ degeneration by the ‘three-joint complex'. Teichtahl et al. investigated the relationship between MCs and intervertebral DD firstly [23]. However, they did not investigate the correlation of MCs with the DD in different grade of endplate defect. We investigate the relationship between endplate defect, size of Modic lesion, and degeneration of 3-joint complex in the lower lumbar spine. Further, we are the first to find the concordance between the presence of FJ degeneration and endplate defect. Our results suggested that the size of Modic change II in Grade 3 endplate defect was significantly larger than other kinds of endplate defect. In addition, the severity of endplate defect in the L4-L5 level is higher than that in other levels.

Kentaro et al. demonstrated an association between endplate defect and FJ degeneration in rheumatoid arthritis [24]. Our data add knowledge for the relationship between endplate defect and FJ-originated lower back pain. We found that there was a significant difference in facet tropism between the four types of endplate defect in patients with LBP. However, there was no difference in facet orientation between the four types of endplate defect. Kerttula et al. demonstrated that MCs type I were related to increasing the size of endplate defects and decreasing the disc height in a 1-year follow-up clinical study. [25]. Further, Feng et al. demonstrated that endplate defects, MCs, and DD were risk factors for episodes of severe LBP [26]. Quantitative measurements showed that the different endplate defects had a negative association with disc signal intensity, disc bulging, and disc height in the adjacent disc. The coexistence of MCs and endplate defects in the vertebral body has been reported in several studies. However, none focused on the relationship between them. Our research found that the four types of endplate defects differed in segment distribution, configuration features, and strength of association with the size of MCs. We found that LBP can be accelerated by the increasing prevalence of endplate defects, MCs, and DD, which was in accordance with previous studies [24, 27, 28]. Moreover, we were surprised to find that endplate defect group 1 (focal) was associated with the highest degree of disc degeneration (DD4). The reason for this phenomenon is that most enrolled patients suffered from MCs II, which resulted in bias. The DD, which was caused by endplate defects, synergies with endplate defects and MCs type II to promote the progress of LBP.

Symptoms of the patients in the four groups showed a trend of significant improvement within 1 week after surgery, but a recessive trend after 24 weeks. We hypothesized that worst pain and functional recovery were associated with severe endplate defects, MCs, DD, and FJ degeneration, all of which were the potential risk factors for the lower back pain. Previous studies had demonstrated that endplate defects would accelerate MCs and DD, which correlated with heritability [24] and vertebral fracture [29]. In addition, we found no significant differences among the four groups in clinical efficiency. We attributed this phenomenon to advantages of percutaneous transforaminal endoscopic discectomy for its less trauma and quicker recovery.

## 5. Limitations

Despite the strengths, several limitations could not be avoided in the present investigation. First, our study could be improved by enrolling a large number of subjects, which could help to accurately investigate the relationship between these degenerative changes. Thus, further large-scale prospective studies with long-term follow-up and normalized measurement are needed. Second, patients with the same type of herniation, which was considered as a confounding factor, should be enrolled in a prospective study.

## 6. Conclusion

Presence of endplate defects may increase the size of MCs I and MCs II in segments with severe DD. However, the size of Modic lesions was not directly associated with clinical symptoms. Endplate defects would accelerate the change of FJ orientation and facet tropism in segments with MCs. In summary, the results of this study highlight the significance of endplate defects and its association with the variation of determinants associated with DD and FJ degeneration. Further, endplate defects may be the initiating factor for MC and intervertebral DD and FJ degeneration. Our study demonstrated that progress in endplate defects resulted in a deterioration in both pain scores and functional disability measures at each follow-up time point from 1 week before surgery to 24 weeks after surgery.

## Figures and Tables

**Table 1:** Types of MC in the lumbar vertebral body according to MRI.

Modic classification: MRI changes and associated pathological features			
Vertebral endplates	T1-weighted sequences	T2-weighted sequences	Histopathology

Modic 1	Hypo-signal	Hyper-signal	Oedema, inflammation

Modic 2	Hyper-signal	Iso-signal or hyper-signal	Fatty changes

Modic 3	Hypo-signal	Hypo-signal	Fibrous process

**Table 2:** Baseline data and intraoperative characteristics of patients in each group.

	Endplate defect 0(n=17)	Endplate defect 1(n=29)	Endplate defect 2(n =12)	Endplate defect 3(n =17)	*P* Value
Demographic characteristics					

Gender(male:female)	14:3	10:19	6:6	8:9	0.019*∗*

Age (years)	57.59(10.03)	56.03(13.16)	50.67(15.23)	57.24(13.36)	0.491

Height (cm)	171.23 (5.23)	164.32 (7.33)	169.50 (6.86)	167.75 (10.13)	0.066

Weight (kg)	74.46 (9.35)	65.30 (11.16)	71.58 (7.35)	67.19 (8.95)	0.04*∗*

BMI distribution (kg/m^2^)					

Normal range: 18.5 to 25	6(46.2)	14(63.6)	6(50)	11(68.8)	0.342

Overweight: 25 to 30	6(46.2)	5(22.7)	6(50)	5(31.2)	

≥30	1(7.7)	3(13.6)	0	0(0)	

Employed (Yes / No)	9: 8	12: 17	6: 6	5: 12	0.263

Duration of LBP (months)	61.79(117.50)	46.71 (74.22)	15.43(15.12)	17.06(27.95)	

Tobacco use, n (%)	6(35.29%)	4(13.79%)	5(41.66%)	5(29.41%)	0.738

Alcohol consumption, n (%)	6(35.29%)	1(3.44%)	1(8.33%)	2(11.76%)	0.402

Hypertension, n (%)	8(47.05%)	7(24.13%)	4(33.33%)	3(17.64%)	0.677

Diabetes mellitus, n (%)	1(5.88%)	5(17.24%)	2(16.66%)	1(5.88%)	0.093

Cardiovascular diseases, n (%)	3(17.64%)	1(3.44%)	1(8.33%)	0(0%)	0.169

Respiratory diseases, n (%)	1(5.88%)	2(6.90%)	1(8.33%)	3(17.65%)	

Type of occupation, n (%)					0.874

Sitting	2(11.7%)	5(17.2%)	3(25%)	2(11.8%)	

Mostly walking	7(41.2%)	13(44.8%)	2(16.7%)	7(41.2%)	

Walking and some lifting	4(23.5%)	6(20.7%)	5(41.7%)	5(29.4%)	

Hard physical work	4(23.5%)	5(17.2%)	2(16.7%)	3(17.6%)	

Level of herniation					0.656

L2/3	0(0%)	2(6.8%)	0(0%)	1(5.9%)	

L3/4	3(17.6%)	2(6.8%)	1(8.3%)	0(0%)	

L4/5	8(47%)	12(41.3%)	7(58.3%)	10(58.8%)	

L5/S1	6(35.2%)	13(44.8%)	4(33.3%)	6(35.2%)	

Clinical outcomes					

Blood loss (ml)	5.69(4.53)	6.04(4.00)	4.58(2.81)	6.38(5.09)	0.715

Weight of nucleus pulposus (g)	2.94(4.53)	3.04(0.92)	3.00(0.95)	3.31(1.03)	0.740

Note: *∗*P < 0.05

**Table 3:** Correlation between lumbar image parameters and clinical outcomes.

	ED	FT(left)	FT(right)	FJOA(left)	FJOA(right)	DD	MCs I	MCs II	ODI	VAS
ED	1	-0.177	-0.141	-0.223	-0.170	0.025	-0.136	0.230*∗*	-0.174	-0.205

FT(left)		1	0.627*∗*	0.060	-0.005	-0.004	0.269	0.204	0.050	0.106

FT(right)			1	0.036	0.141	0.120	0.296	0.048	-0.016	-0.043

FJOA(left)				1	0.581*∗*	0.160	0.227	0.136	0.074	0.033

FJOA(right)					1	0.230*∗*	-0.027	0.112	0.028	-0.106

DD						1	0.621	0.079	-0.045	-0.119

MCs I							1	0.220	0.050	-0.159

MCs II								1	-0.107	-0.016

ODI									1	0.740*∗*

VAS										1

Note: *∗*P < 0.05

Abbreviations: ED: endplate defect; FT: facet tropism; FJOA: facet joint osteoarthritis grades; DD: disc degeneration; MCs: Modic changes; ODI: Oswestry Disability Index; VAS: Visual Analogue Scale

**Table 4:** Clinical outcomes before and after surgery at different time points.

	Endplate defect 0	Endplate defect 1	Endplate defect 2	Endplate defect 3
VAS score				

Before surgery				

1 week after surgery	2.18(2.35) *∗*	3.43(2.67) *∗*	2.27(3.00) *∗*	2.06(2.25) *∗*

2 weeks after surgery	1.94(2.36) *∗*	2.79(2.47) *∗*	2.09(2.95) *∗*	1.41(1.87) *∗*

4 weeks after surgery	1.53(2.35) *∗*	2.54(2.57) *∗*	1.64(2.66) *∗*	1.06(1.48) *∗*

8 weeks after surgery	1.65(2.62) *∗*	1.89(2.36) *∗*	1.45(2.66) *∗*	0.88(1.41) *∗*

12 weeks after surgery	1.59(2.43) *∗*	1.61(2.36) *∗*	1.36(2.66) *∗*	0.59(1.28) *∗*

24 weeks after surgery	1.59(2.48) *∗*	1.82(2.47) *∗*	1.18(2.56) *∗*	0.41(1.06) *∗*

ODI score				

Before surgery				

1 week after surgery	8.15(8.73) *∗*	12.79(12.63) *∗*	13.65(14.16) *∗*	7.41(8.17) *∗*

2 weeks after surgery	6.46(8.13) *∗*	11.36(11.71) *∗*	13.20(14.12) *∗*	6.46(7.82) *∗*

4 weeks after surgery	6.99(8.68) *∗*	10.68(11.35) *∗*	11.85(12.83) *∗*	5.51(7.52) *∗*

8 weeks after surgery	5.61(8.37) *∗*	8.32(10.78) *∗*	9.90(11.95) *∗*	3.92(5.10) *∗*

12 weeks after surgery	5.72(8.82) *∗*	9.06(12.17) *∗*	9.00(12.09) *∗*	2.33(4.16) *∗*

24 weeks after surgery	5.82(9.06) *∗*	8.94(12.48) *∗*	8.25(11.34) *∗*	1.27(2.45) *∗*

Note: *∗*P < 0.05 vs. pre-surgery

Abbreviations: VAS: visual analog scale; ODI: Oswestry Disability Index.

## Data Availability

The data used to support the findings of this study are included within the article.
